# Effect of Rapid High-Intensity Light-Curing on Increasing Transdentinal Temperature and Cell Viability: An In Vitro Study

**DOI:** 10.3390/polym16111466

**Published:** 2024-05-22

**Authors:** Samille Biasi Miranda, Rodrigo Barros Esteves Lins, Marina Rodrigues Santi, Giovanna Corrêa Denucci, Cleyton Cézar Souto Silva, Silvana de Fátima Ferreira da Silva, Daniela de Araújo Viana Marques, Marcos Antônio Japiassú Resende Montes

**Affiliations:** 1 School of Dentistry, University of Pernambuco, Recife 50100-130, Pernambuco, Brazil; 2 School of Dentistry, Federal University of Alagoas, Maceió 57072-900, Alagoas, Brazil; rodrigo.lins@foufal.ufal.br; 3 Piracicaba Dental School, University of Campinas, Piracicaba 13414-018, São Paulo, Brazil; marina.rsanti@gmail.com; 4 Department of Cariology and Operative Dentistry, Indiana University School of Dentistry, Indianapolis, IN 46202, USA; gdenucci@iu.edu; 5 Department of Clinical Nurse, Federal University of Paraiba, João Pessoa 58058-600, Paraíba, Brazil; cleyton.souto@academico.ufpb.br; 6 School of Biological Sciences, University of Pernambuco, Recife 50100-130, Pernambuco, Brazil; silvana.ferreira@upe.br (S.d.F.F.d.S.); daniela.viana@upe.br (D.d.A.V.M.); 7 Department of Dental Materials, University of Pernambuco, Recife 50100-130, Pernambuco, Brazil; marcos.japiassu@upe.br

**Keywords:** bulk-fill composite resin, polymerization, high irradiance, temperature, cell viability

## Abstract

Background: This study investigated effects of rapid high-intensity light-curing (3 s) on increasing transdentinal temperature and cell viability. Methods: A total of 40 dentin discs (0.5 mm) obtained from human molars were prepared, included in artificial pulp chambers (4.5 × 5 mm), and subjected to four light-curing protocols (n = 5), with a Valo Grand light curing unit: (i) 10 s protocol with a moderate intensity of 1000 mW/cm^2^ (Valo-10 s); (ii) 3 s protocol with a high intensity of 3200 mW/cm^2^ (Valo-3 s); (iii) adhesive system + Filtek Bulk-Fill Flow bulk-fill composite resin in 10 s (FBF-10 s); (iv) adhesive system + Tetric PowerFlow bulk-fill composite resin in 3 s (TPF-3 s). Transdentinal temperature changes were recorded with a type K thermocouple. Cell viability was assessed using the MTT assay. Data were analyzed using one-way ANOVA and Tukey tests for comparison between experimental groups (*p* < 0.05). Results: The 3 s high-intensity light-curing protocol generated a higher temperature than the 10 s moderate-intensity standard (*p* < 0.001). The Valo-10 s and Valo-3 s groups demonstrated greater cell viability than the FBF-10s and TPF-3 s groups and statistical differences were observed between the Valo-3 s and FBF-10 s groups (*p* = 0.023) and Valo-3 s and TPF-3 s (*p* = 0.025), with a potential cytotoxic effect for the FBF-10 s and TPF-3 s groups. Conclusions: The 3 s rapid high-intensity light-curing protocol of bulk-fill composite resins caused a temperature increase greater than 10 s and showed cell viability similar to and comparable to the standard protocol.

## 1. Introduction

Aiming to simplify the restorative protocol, bulk-fill composite resins were created. These resins allow for the insertion of a single increment up to 5 mm in thickness [1], making clinical handling easier and reducing the sensitivity of the incremental technique [2]. Bulk-fill composite resins have been shown to be superior to conventional composite resins in terms of lower polymerization shrinkage [3], greater marginal integrity [4], and greater depth of polymerization [5].

The evolution of the light-curing protocol has been influenced by the constant search to improve the restorative procedure [6]. Currently, the trend in light-curing development has been to increase emitted light intensity to decrease exposure time [7]. Ultra-fast light-curing was introduced with high levels of radiant emittance (up to 3000–3500 mW/cm^2^) and shortened exposure time [8]. However, the use of very short exposure times (1–3 s) has been shown to be insufficient to adequately polymerize restorative materials, leading to the need to formulate a material compatible with this technique [9].

Thus, manufacturers of dental materials have modified the composition of current bulk-fill composite resin to enable light-curing in just 3 s with a radiant light emittance of 3050 mW/cm^2^ [10]. The new bulk-fill composite resins present a fundamentally altered polymerization process [11], where shorter polymer chains and a more homogeneous structure are generated, enabling better mechanical properties [12] and ensuring its use in short times of exposure to light with high radiant exposure, without adverse consequences [6,8,9].

However, high irradiance values have become a concern due to their potential risk to soft tissues and dental pulp [13], especially because the thickness of remaining dentin, the composition of the composite resin, the technique of insertion into the cavity and the distance between the light source and the restorative material are factors that directly affect the increase in intrapulpal temperature [14]. It is vital to ensure pulp health, as any substantial increase in temperature could lead to irreversible damage to the dentin–pulp complex [15]. Since bulk-fill composite resins are applied in deep cavities, the heat generated during light-curing is transferred into the cavity, especially with high-intensity protocols [16]. Furthermore, the cellular response of restorative materials recently introduced into the dental market is of clinical importance, as their clinical success depends on their biological compatibility [17].

The assessment of toxicity induced by restorative materials is necessary to elucidate the potential risks to the patient’s health, especially considering the relationship between the release of residual unpolymerized monomers from the resin matrix and the potential cytotoxic effects over time [18]. These substances can diffuse into the dentin and reach the dental pulp [19], causing adverse reactions in the pulp tissues, ranging from post-operative sensitivity to irreversible pulp damage [20].

In clinical practice, time optimization is achieved by shortening the light-curing time of bulk-fill composite resins to 3 s. However, it is important to evaluate the thermal and biological changes resulting from this light-curing protocol in vitro to ensure that it does not compromise polymerization or induce any undesirable side effects. The aim of this study was to compare the effects of different bulk-fill composite resins and light-curing modes on transdentinal temperature and cell viability. The null hypotheses were: (i) There is no significant difference in the increase of transdentinal temperature between the high-intensity (3 s) and standard (10 s) light-curing protocols of bulk-fill composite resins. (ii) The different light-curing protocols of bulk-fill composite resins do not appear to affect cell viability.

## 2. Materials and Methods

The bulk-fill composite resins Tetric PowerFlow (Ivoclar Vivadent, Schaan, Liechtenstein) and Filtek Bulk-Fill Flow (3M ESPE, St. Paul, MN, USA) were subjected to two light-curing protocols using the Valo Grand Cordless (Ultradent, South Jordan, UT, USA). The protocols included ultra-fast light-curing for 3 s with a high radiant emittance of 3200 mW/cm^2^ and standard light-curing for 10 s with a moderate radiant emittance of 1000 mW/cm^2^. Table 1 presents the characteristics of the materials, while Table 2 provides details on the light-curing protocols.

### 2.1. Obtaining Dentin Discs

The protocol for obtaining dentin discs was approved by the Human Research Ethics Committee of the University of Pernambuco (CEP) in accordance with resolution no. 466/12 of the National Health Council (number: 66493323.6.0000.5207). Forty sound, human molars, without cavities, restorations, cracks, fissures, or fractures, were selected for this research. The sample size was determined based on previous experiments with similar characteristics and outcomes, regarding the increase in temperature during light-curing [21,22,23,24] and cell viability analysis [25,26,27]. The recommendation from the risk of bias (RoB) tool for laboratory studies on dental materials [28] was followed. A single blind operator randomly allocated the teeth into four experimental groups (n = 5) based on the type of bulk-fill composite resin and light-curing protocol. Table 3 presents the groups and tests performed.

**Figure 1:**
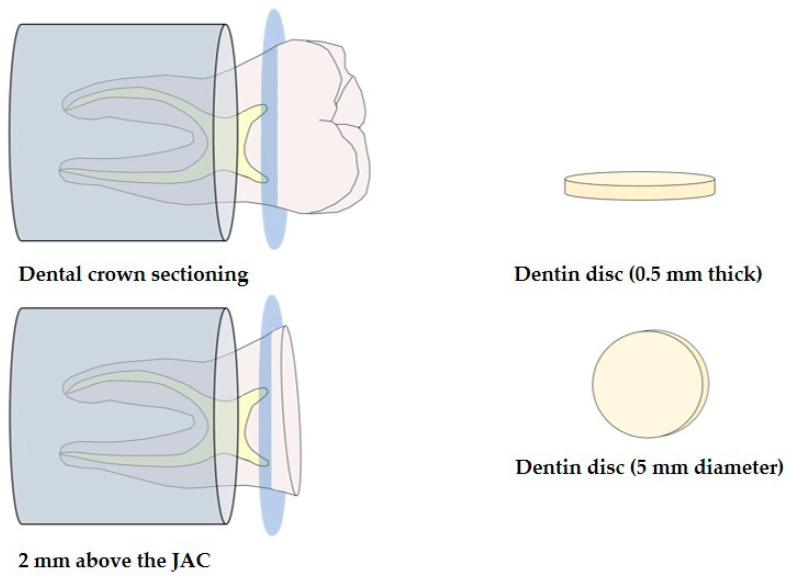
Obtaining dentin discs.

The dental elements were secured in PVC cylinders filled with chemically activated acrylic resin and positioned with the occlusal surface facing upward. The dental crown was transversely sectioned in the mid-coronal region, 2 mm above the cementoenamel junction, parallel to the occlusal surface and 0.5 mm above the roof of the pulp chamber, to obtain deep dentin discs. A precision cutting machine (Isomet 1000, Buehler, Lake Bluff, IL, USA) with a double-sided diamond disc (Isomet Diamond Wafering Blades-Buehler, Lake Bluff, IL, USA) under constant irrigation was used for this purpose. The thickness was measured using a digital caliper with an accuracy of 0.01 mm (KALA, Curitiba, Paraná, Brazil). Next, excess material was manually removed using silicon carbide abrasive sandpaper #400 and #600 grit (Buehler Ltd., Lake Bluff, IL, USA) to achieve a thickness of 0.5 mm. This process also helped to regulate the surface finish and remove any impurities. A flat cylindrical diamond bur #2135F (KG, Sorensen, Cotia, São Paulo, Brazil) was used to standardize the dentin disc into a circular shape with a diameter of 5 mm, as shown in Figure 1. The discs were prepared by a single trained and calibrated operator. After rinsing the samples for 30 s with deionized water, they were stored in phosphate buffer solution (PBS, pH 7.2).

### 2.2. Artificial Pulp Chamber

**Figure 2:**
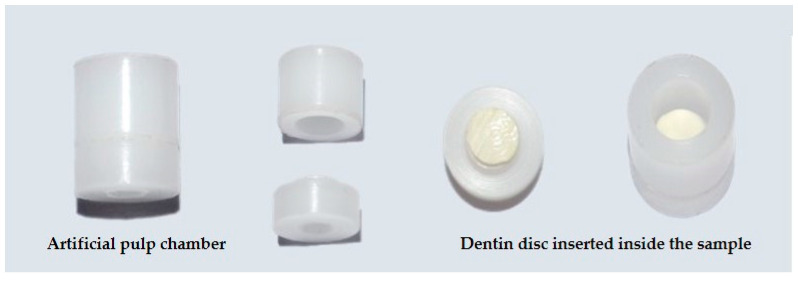
Artificial pulp chamber device.

Cylindrical Teflon samples with insertions were made to include the dentin discs, simulating the dimensions of a posterior tooth with little remanent dentin. The internal measurements were 4.5 mm × 5 mm (Odeme Dental Research, Santa Catarina, Brazil). The dentin discs were positioned with the occlusal surface facing upwards and the pulpal surface facing downwards. They were then inserted into the lower part of the device (support for the disc), and the upper part was fitted to stabilize the assembly (Figure 2). The cylindrical samples and dentin discs underwent sterilization with gamma radiation (25 kGy) for 10 h and 37 min to eliminate microorganisms.

For groups Valo-10 s and Valo-3 s, the dentin disc was inserted into the artificial pulp chamber device and the tip of the light curing unit (LCU) was positioned 0 mm from the surface of the set, with both light-curing protocols being applied (10 and 3 s). Groups FBF-10 s and TPF-3 s simulated the restorative protocol with Single Bond Universal adhesive system (3M ESPE, St. Paul, MN, USA) applied according to the manufacturer’s instructions on the occlusal surface of the dry dentin disc, with the aid of a microbrush applicator (KG, Sorensen, Cotia, São Paulo, Brazil), frictioning for 20 s, drying with an air jet for 5 s and light curing for 10 s with the Valo Grand LCU (Ultradent, South Jordan, UT, USA) with an intensity of light of 1000 mW/cm^2^. Then, the device was filled by a single operator with bulk-fill composite resins (Filtek Bulk-Fill Flow and Tetric PowerFlow) on the adhesive system with the aid of the applicator tip in a single increment (4 mm) and light-cured according to the standard (10 s) and high-intensity (3 s) light-curing protocols.

### 2.3. Assessment of Transdentinal Temperature Changes

Temperature changes were recorded using a digital thermometer coupled to a type K thermocouple with a datalogger (Instrutherm, São Paulo, São Paulo, Brazil) with a resolution of 0.1 °C. The changes due to light intensity emitted by the LCU were measured during the restorative protocol [1,14,29].

To measure the temperature increase caused by the light intensity emitted by the LCU, the type K thermocouple wire was inserted into the lower region of the artificial pulp chamber device, below the pulp surface of the dentin disc. To assess the rise in temperature in the dentin disc, a thermocouple detector was placed beneath the pulpal surface, and the temperature resulting from the light-curing of the bulk-fill composite resins (Tetric PowerFlow and Filtek Bulk-Fill Flow) was measured.

**Figure 3:**
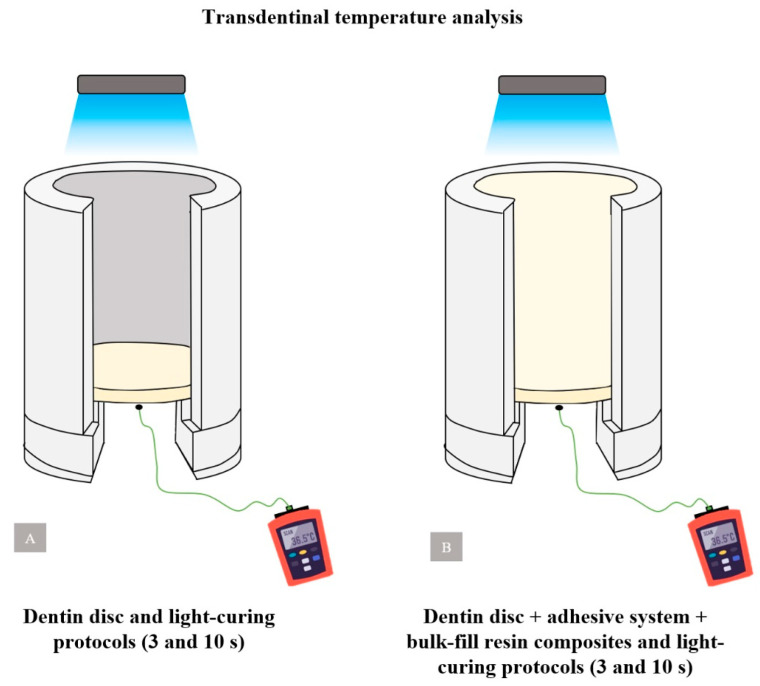
Assessment of transdentinal temperature. (**A**) Experimental groups Valo-10 s and Valo-3 s; (**B**) Experimental groups FBF-10 s and TPF-3 s.

For all evaluations, the tip of the LCU was positioned in contact (0 mm) with the upper surface of the artificial pulp chamber. The temperature increase in all samples was determined in an identical and controlled temperature environment (27 °C), by a single trained and calibrated operator. The temperature was recorded in each sample according to the group, immediately after the two light-curing protocols (3 and 10 s). The average change in transdentinal temperature was obtained through the difference between the ambient temperature and the temperature recorded after the experiments. Figure 3 illustrates this analysis.

### 2.4. Cell Viability Analysis

#### 2.4.1. Cell Cultivation

The Vero cell line (CCL-81, Rio de Janeiro, Brazil) originating from the normal epithelium of the green monkey renal cortex was seeded at a concentration of 5 × 10^5^ cells/mL in a 25 cm^2^ cell culture bottle containing RPMI-1640 medium supplemented with 10% fetal bovine serum (FBS), 2 mM glutamine, 100 units/mL penicillin, and 100 µg/mL streptomycin. The cells were incubated in a humidified incubator at 37 °C and 5% CO_2_. The medium was changed every 48 h, and the cells were maintained until a monolayer was formed. To maintain the cells, the monolayer cell culture was washed with RPMI-1640 without SBF and then treated with trypsin for cell dissociation. After the trypsinization process, the cells were quantified in the Neubauer chamber and cultured in 25 cm^2^ culture bottles at a density of 2 × 10^5^ cells per bottle.

#### 2.4.2. MTT Analysis

**Figure 4:**
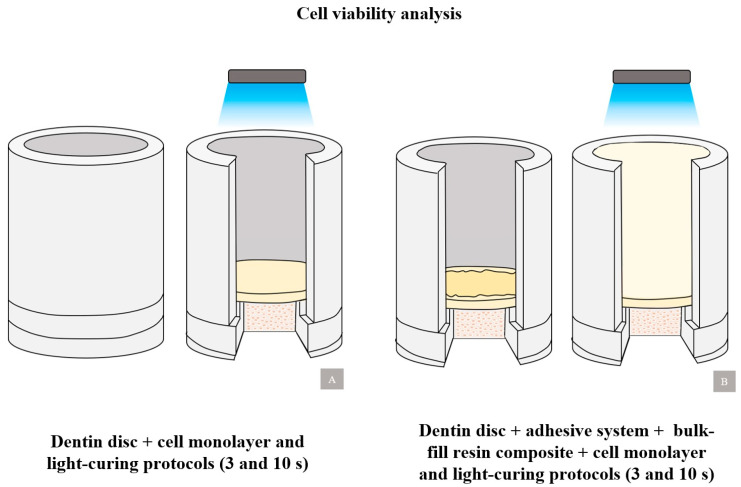
Dentin barrier method for MTT analysis. (**A**) Experimental groups Valo-10 s and Valo-3 s; (**B**) Experimental groups FBF-10 s and TPF-3 s.

Cytotoxicity analyses were performed following the protocol established by Mosmann (1983) [30] and the ISO 10993-5:2009 standard [31]. The number of Vero lineage cells was determined by counting in a Neubauer chamber and adjusted to a concentration of 1 × 10^6^ cells/mL in RPMI-1640 medium with 10% FBS. The cells were then incubated for 48 h at 37 °C and 5% CO_2_. After incubation, the cells underwent tests based on the experimental groups (Figure 4) in 12-well culture plates using the dentin barrier method. The artificial pulp chamber device was positioned so that the pulpal face of the dentin disc was in direct contact with the cell culture medium.

Then, 10μL of MTT (3-(4,5-Dimethylthiazol-2-yl)-2,5-Diphenyltetrazolium Bromide) (0.5 mg/mL) was added, and the cultures were incubated for an additional 3 h. The supernatant was removed, and the pellet was solubilized in a 96-well culture plate with 100 μL of DMSO. The precipitate resulting from MTT reduction was quantified spectrophotometrically at 570 nm. Wells containing only MTT and DMSO were used as blanks. The negative control consisted of cells treated with culture medium only, resulting in 100% cell viability. Viability was determined by calculating the average absorbances obtained in the tests divided by the average absorbances of the controls multiplied by 100. The experiments were conducted in quintuplicate, following the recommendation of at least three replicates for in vitro cytotoxicity tests and the degree of cytotoxicity of the material was analyzed under different conditions and classified according to ISO 10993-5:2009 [31]. A reduction in cell viability of more than 30% indicates a cytotoxic effect of the restorative material.

### 2.5. Statistical Analysis

Normality and homogeneity of variance of the data were assessed using Shapiro–Wilk and Levene tests, respectively. The changes in the transdentinal temperature and cell viability were analyzed using one-way ANOVA and Tukey tests, the differences between the groups were analyzed based on different restorative materials and light-curing protocols, with a significance level of 5%.

## 3. Results

### 3.1. Increased Transdentinal Temperature

Table 4 shows the temperature difference (ΔT) recorded during the light-curing protocols in the two different modes at different time intervals (3 and 10 s). It was possible to observe that in groups Valo-10 s and FBF-10 s, where the artificial pulp chamber device was exposed to light with the moderate-intensity light-curing protocol (10 s), the values referring to the temperature increase were lower [Valo-10 s (7.04 °C); FBF-10 s (5.52 °C)] when compared to groups Valo-3 s and TPF-3 s, where high-intensity light (3 s) was applied [Valo-3 s (11.70 °C); TPF-3 s (9.16 °C)]. The greatest temperature variation was recorded in group Valo-3 s, followed by group TPF-3 s (different from each other [*p* = 0.007] and from the other groups [*p* < 0.026]), demonstrating that there was greater temperature increase for groups where the high-intensity protocol was used (3 s).

### 3.2. Cell Viability Analysis

Table 5 presents the cell viability results (%) of Vero cells exposed to light-curing protocols using the dentin barrier method. The results show that only groups FBF-10 s and TPF-3 s had cytotoxic effects, with a reduction in cell viability of more than 30% compared to the negative control, at 69.27% and 69.50%, respectively.

In groups Valo-10 s and Valo-3 s, where light was applied directly to the dentin disc, higher percentages of cell viability were found, when compared to groups FBF-10 s and TPF-3 s, which received the adhesive system and bulk-fill composite resins [Valo-10 s (73.79%); Valo-3 s (88.09%); FBF-10 s (69.27%); TPF-3 s (69.50%)]. When compared with each other, statistical differences were found only between groups Valo-3 s and FBF-10 s (*p* = 0.023), and between groups Valo-3 s and TPF-3 s (*p* = 0.025), where it is possible to observe that during exposure to high-intensity light (3 s), there was greater cell inhibition for the group with the adhesive system and Tetric PowerFlow bulk-fill composite resin.

## 4. Discussion

Short light-curing times optimize clinical time for professionals, increase patient comfort, and reduce the risk of contamination [32]. The results of our study show that in a simulated clinical condition of a posterior tooth with little remanent dentin, there was a greater increase in transdentinal temperature during the high-intensity protocol (3 s) compared to the standard protocol (10 s). Therefore, we reject the first hypothesis. However, both light-curing modes showed a similar effect on cell viability, indicating a potential cytotoxic effect. Therefore, the second hypothesis was accepted.

The increase in intrapulpal temperature during light-curing can be caused by factors related to the light source (intensity, wavelength, and exposure time), the restorative material (density, thermal conductivity and capacity, and increment thickness), and the tooth (thickness of remanent dentin, dentin shade, and type of dental element) [33,34]. Heat released by the exothermic reaction of the restorative material and exposure to light-curing unit [35] can also contribute to pulp damage. This is particularly concerning as the pulp is a highly vascularized tissue with the ability to dissipate heat to the dentin–pulp complex [14].

The exothermic reaction can be a concern when using bulk-fill composite resins because of the larger volume of resin that needs to be light-cured simultaneously [21]. Research has shown a proportional relationship between the increase in temperature and the amount of restorative material [21,36]. In this study, we exclusively used flowable bulk-fill composite resins, since there is consensus in the literature that flowable composite resins generate a more intense exothermic reaction during polymerization than non-flowable composite resins, generating a more intense exothermic reaction [14,16,22,37].

An in vitro study reported a significant increase in temperature ranging from 1.5 to 23.2 °C in the pulp chamber, which is directly influenced by the high-intensity light during light-curing [38]. Although controversial, some researchers have determined that an intrapulpal temperature increase of 5.5 to 5.6 °C would be the critical threshold for irreversible pulp damage [15,21,39]. Although, in vivo evaluations of human teeth have shown that average temperature increases of 11.2 °C do not cause pulp damage [40].

The increase in transdentinal temperature in this study was high enough to exceed the critical temperature where pulpal damage could begin in the restorative materials, demonstrating that the change in temperature was proportional to the increase in light intensity, corroborating with previous studies [16,41,42,43]. Our findings report a rise of 9.16 °C during the 3 s light-curing of Tetric PowerFlow bulk-fill composite resin, with results similar to a previous study that recorded an increase between 10.8 and 12 °C [32].

Our results may have been influenced by the 0.5 mm thickness of remanent dentin. This thin layer of dentin may be insufficient to prevent temperature increase and damage to the pulp, this is supported by a study that observed an increase in intrapulpal temperature in all samples when decreasing the dentin thickness from 1 mm to 0.5 mm [14]. Therefore, the 3 s protocol is considered thermally safe as long as the tooth has sufficient dentin thickness [44]. Clinicians should consider using a smaller volume of resin in the initial layer of the restoration to minimize thermal irritation to pulp tissue for very deep cavities [37].

The authors are not aware of any previous studies that have evaluated the cytotoxicity of bulk-fill composite resins designed for 3 s light-curing through the dentin barrier test, with respect to cell viability. The utilization of this method renders laboratory evaluations more relevant, particularly by simulating the clinical conditions to which the materials are exposed in the oral cavity [17]. This allows for the analysis of the chemical toxicity of the pulp tissue through the dentin, reflecting the in vivo conditions with greater precision [45]. Our study shows that different types of bulk-fill composite resin and light-curing protocols have a potential cytotoxic effect that exceeds the inhibition limit value recommended by ISO 10993-5 [31].

Toxicity risks are a concern for materials that come into contact with oral tissues [46]. The cytotoxic effects of resinous dental materials depend on the concentration of leachable monomers, especially the release of residual monomers that have not polymerized adequately [47], which can diffuse into the dentin and reach the dental pulp, affecting pulp vitality [48]. Additionally, the thickness of dentin is closely related to the diffusion of substances to the pulp through the dentinal tubules [49]. It is important to note that in bulk-fill composite resins, greater irradiation depth results in greater cytotoxicity, particularly in increments greater than 4 mm [50]. This may be associated with our results, as we applied a 4 mm thickness in all experimental tests.

A potential explanation for the cytotoxic effect observed in two experimental groups may be attributed to the use of the universal adhesive system in our study. This system involves the application of primer and acid monomer in a single step, which demineralizes and infiltrates the substrate simultaneously [51]. These additional components have the potential to alter the behavior of the dentin–pulp complex, where monomers may be released after polymerization [52]. Diffusion through the dentinal tubules can reflect on cell viability and potentially cause irreversible damage to the pulp tissue [53]. Monomers such as HEMA and Bis-GMA, which are considered toxic substances, can cause harm in deep cavities that are in direct contact with the pulp tissue [54].

The application of resinous materials in deep dentin remains a challenge [55]. This study found no difference in cytotoxicity between different light-curing times, which is consistent with a previous study that evaluated the cytotoxicity of adhesive systems [56]. Unreacted monomers can cause cytotoxicity problems, such as irritation of the pulp and soft tissues around restorations [57]. The amount of remnant dentin also seems to have influenced this aspect; a greater thickness of dentin could prevent or reduce the amount of residual monomers capable of causing damage to the pulp [17].

With knowledge of the limitations, the experimental conditions for this in vitro study were standardized and controlled. The dentin discs were prepared with a smooth surface to ensure adequate adhesion. Factors such as the distance of 0 mm from the tip of the LCU and control of the device’s irradiance would be difficult to replicate in a real clinical situation. The artificial pulp chamber used in the samples may have different heat dissipation potential compared to the vital tooth. Temperature was measured at a single point, so it would be beneficial to evaluate other regions; it would also be helpful to incorporate blood circulation and adjust the basal temperature to 37 °C. Furthermore, studies with cells from dental pulp would be closer to clinical reality. We recommend conducting clinical trials to confirm our results more precisely. However, this study presents a thermal and biological analysis of bulk-fill composite resins that are specifically designed for 3-s high-intensity light-curing. This study helps to establish a standardized protocol for safe use by professionals in clinical practice.

## 5. Conclusions

The rapid high-intensity light-curing protocol (3 s) resulted in a greater increase in temperature compared to the moderate-intensity standard (10 s), indicating a proportional relationship between temperature and intensity. However, both 3 s and 10 s light-curing protocols showed similar and comparable cell viability. It is recommended that clinicians use the rapid high-intensity protocol with caution in teeth with little remnant dentin.

## Figures and Tables

**Table 1:** Tested materials, classifications, and compositions.

Material	Type, Color	Manufacturer	Composition
Single Bond Universal	One-step universal adhesive system	3M ESPE, St. Paul, EUA	MDP, phosphate monomer, dimethacrylate, HEMA, Bis-GMA, copolymerizer, dimethylaminobenzoate, vitrabond, ethanol, water, silane, and primer.
Filtek Bulk-Fill Flow	Bulk-Fill Flow Resin, A2	3M ESPE, St. Paul, EUA	Bis-EMA, Bis-GMA, UDMA, treated silanized ceramic, Benzotriazole, substituted dimethacrylate, TEGDMA, and ytterbium fluoride.
Tetric PowerFill	Bulk-Fill Flow Resin, IVA	Ivoclar Vivadent AG, Schaan, Liechtenstein	Dimethacrylates, barium glass, ytterbium trifluoride, and copolymers.

Legend: MDP, 10-methacryloyloxidecamethylene phosphoric acid; HEMA, 2-hydroxyethyl methacrylate; Bis-GMA, bisphenol-A glycidyl methacrylate; Bis-Ema, bisphenol hydroxyethyl methacrylate; UDMA, urethane dimethacrylate; and TEGDMA, triethylene glycol dimethacrylate.

**Table 2:** Light-curing unit and light-curing protocols.

Light-Curing Unit	Emission Spectrum	Mode	Time	Irradiance (mW/cm^2^)	Manufacturer
Valo Grand	Polywave	Xtra	10 s	1000	Ultradent, South Jordan, EUA
		Standard	3 s	3200	

**Table 3:** Experimental design in different treatment groups.

Group	n	Procedure	Light-Curing
Valo-10 s	5	Artificial pulp chamber + dentin disc	10 s, 1000 mW/cm^2^
Valo-3 s	5	Artificial pulp chamber + dentin disc	3 s, 3200 mW/cm^2^
FBF-10 s	5	Artificial pulp chamber + dentin disc + adhesive system + Filtek Bulk-Fill Flow	10 s, 1000 mW/cm^2^
TPF-3 s	5	Artificial pulp chamber + dentin disc + adhesive system + Tetric PowerFlow	3 s, 3200 mW/cm^2^

**Table 4:** Mean and standard deviation (SD) of the difference in transdentinal temperature (ΔT-°C) in different light-curing protocols.

Group	Transdentinal Temperature (ΔT-°C)
Valo-10 s	7.04 (1.4) C
Valo-3 s	11.70 (0.5) A
FBF-10 s	5.52 (1.3) C
TPF-3 s	9.16 (0.7) B

Means and standard deviation followed by different letters indicate statistical differences according to one-way ANOVA and Tukey tests, with significant level set at 5%. Uppercase letters compare groups of treatment. n = 5.

**Table 5:** Cell viability (Mean and SD) determined by the MTT assay in different light-curing protocols.

Group	Cell Viability (%)
Valo-10 s	73.79 (6.8) AB
Valo-3 s	88.09 (5.5) A
FBF-10 s	69.27 (10.0) B
TPF-3 s	69.50 (12.5) B

Means and standard deviation followed by different letters indicate statistical differences according to one-way ANOVA and Tukey tests, with significant level set at 5%. Uppercase letters compare groups of treatment. n = 5.

## Data Availability

All data are available within the manuscript.
